# Gut feelings and the reaction to perceived inequity: The interplay between bodily responses, regulation, and perception shapes the rejection of unfair offers on the ultimatum game

**DOI:** 10.3758/s13415-012-0092-z

**Published:** 2012-05-23

**Authors:** Barnaby D. Dunn, Davy Evans, Dasha Makarova, Josh White, Luke Clark

**Affiliations:** 1 Mood Disorders Centre, University of Exeter, Perry Road, EX4 4QG Exeter, UK; 2 Medical Research Council Cognition and Brain Sciences Unit, 15 Chaucer Road, CB2 7EF Cambridge, England UK; 3 University of Cambridge, Cambridge, England UK

**Keywords:** Decision-making, Embodied cognition, Emotion

## Abstract

It has been robustly demonstrated using the ultimatum game (UG) that individuals frequently reject unfair financial offers even if this results in a personal cost. One influential hypothesis for these rejections is that they reflect an emotional reaction to unfairness that overrides purely economic decision processes. In the present study, we examined whether the interplay between bodily responses, bodily regulation, and bodily perception (“interoception”) contributes to emotionally driven rejection behavior on the UG. Offering support for bodily feedback theories, interoceptive accuracy moderated the relationship between changes in electrodermal activity to proposals and the behavioral rejection of such offers. Larger electrodermal responses to rejected relative to accepted offers predicted greater rejection in those with accurate interoception but were unrelated to rejection in those with poor interoception. Although cardiovascular responses during the offer period were unrelated to rejection rates, greater resting heart rate variability (linked to trait emotion regulation capacity) predicted reduced rejection rates of offers. These findings help clarify individual differences in reactions to perceived unfairness, support previous emotion regulation deficit accounts of rejection behavior, and suggest that the perception and regulation of bodily based emotional biasing signals (“gut feelings”) partly shape financial decision making on the UG.

Anyone who has endured the pain of being unreasonably overlooked for promotion, reacted angrily in response to a below-market value offer for their house, or felt slighted by an unduly small pay raise will acknowledge that humans are highly attuned to violations in fairness. Particularly in the financial domain, we are often forced to weigh up the demands of maintaining social equity versus economic self-interest, and how we respond to such dilemmas can have marked economic, social, and personal consequences. It is therefore important to understand the psychological mechanisms that underpin how we respond to perceived unfairness.

The ultimatum game (UG) neatly models the balancing act between financial self-interest and social equity (see Guth, Schmittberger, & Schwarze, 1982). On each trial, a proposer makes a once-only offer of how to divide a sum of money, and the responder either rejects or accepts the proposed division. If the offer is rejected, neither player receives any money. If the offer is accepted, the proposal is implemented. Since it is a one-off offer with no impact on reputation, the “rational” responder behavior is to accept all offers, no matter how unfair. After all, some money is better than no money. However, a proportion of unfair offers are reliably rejected, despite the fact this entails a financial loss for the responder (see, e.g., Sanfey Rilling, Aronson, Nystrom, & Cohen, 2003).

One proposed explanation of this rejection behavior is a failure of emotion regulation. Emotional experience in the face of unfairness (e.g., an increase in anger, disgust, surprise, or a general sense of arousal) is believed to override the economically “rational” response of accepting whatever is offered.^1^ This perspective mirrors a range of work examining the causes and consequences of impaired emotion regulation (Gross, 1998; for a review, see Gross, 2006) and is supported by an array of data on the UG. Inducing a negative mood increases rejection rates (Harle & Sanfey, 2007). Increased activity in the right anterior insula—a brain area implicated in emotion processing and experience—predicts greater rejection rates (Sanfey et al., 2003). When accepting unfair offers, greater activation is seen in ventrolateral prefrontal cortex—a region associated with emotion regulation capability (Tabibnia, Satpute, & Lieberman, 2008). Participants retrospectively report feeling stronger emotions (e.g., anger) to unfair than to fair offers (Pillutla & Murninghan, 1996). Patients with frontal lobe lesions, who often show difficulties in emotion regulation, exhibit elevated rejection rates (Koenigs & Tranel, 2007; although see Moretti, Dragone, & Pellegrino, 2009). Depletion of the neurotransmitter serotonin, which is implicated in emotion regulation, leads to increased rejection after unfair offers (Crockett, Clark, Tabibnia, Lieberman, & Robbins, 2008). Finally, encouraging individuals to adopt emotional reappraisal (an adaptive form of emotion regulation) decreases rejection rates, relative to both emotion suppression and no intervention control conditions (van’t Wout, Chang, & Sanfey, 2010).

It is increasingly realized that substantial individual differences exist in behavioral reactions to perceived unfairness on the UG (see, e.g., Dunn, Makarova, Evans, & Clark, 2010b). There is also evidence of heterogeneity in insular reactivity to unfairness (Kirk, Downar, & Montague, 2011). Experienced Buddhist meditators were found to be less likely to reject unfair offers and also showed a shift in activation during unfair offers from anterior to posterior insula, as compared with a nonmeditating control group. This suggests that emotion regulation mechanisms are not acting uniformly across individuals. The primary aim of the present study was to investigate one possible source of individual variability in emotionally driven rejection behavior.

An important component of emotion is the physiological changes that occur in the body—for example, the experience of butterflies in the stomach during fear. According to bodily feedback theories, these peripheral responses should play a causal role in how we think and feel. James (1884) famously asserted that emotional experience is the perception of changes that occur in the body. These ideas were extended to the decision-making domain in the somatic marker hypothesis (Damasio, 1994; Dunn, Dalgleish, & Lawrence, 2006), which proposes that bodily based emotional biasing signals shape our choices between alternative options. Although the strong claim that bodily response profiles can uniquely differentiate between distinct emotion states (e.g., anger vs. disgust) has not been widely supported (Larsen Bernston, Poehlmann, Ito, & Cacioppo, 2007), there is increasing evidence that bodily feedback contributes to a crude sense of emotional arousal that can influence decision making and is a base component of more nuanced emotion states (see Barrett, Quigley, Bliss-Moreau, & Aronson 2004; Dalgleish, Dunn, & Mobbs, 2009; Dunn et al., 2010a, Study 1). This evidence fits well with the view that distinct emotions such as fear, disgust, and happiness do not actually form “natural kinds” and instead reflect blends of core dimensions of arousal and valence (see Barrett, 2006; Russell & Barrett, 1999).

There is encouraging circumstantial evidence that variation in bodily responses can partly account for individual differences on the UG. Elevated electrodermal (EDA) increases (a measure of autonomic nervous system function) have been shown to unfair, relative to fair, offers on the UG (van’t Wout, Kahn, Sanfey, & Aleman, 2006; Moretti et al., 2009; although see Osumi & Ohira 2009). Moreover, greater heart rate (HR) decelerations have been exhibited to rejected, relative to accepted, offers on the UG (Osumi & Ohira, 2009). These bodily responses are to some extent correlated with UG rejection behavior (Osumi & Ohira, 2009; van’t Wout et al., 2006). Moreover, the right anterior insula region activated during rejections in fMRI studies of the UG is also strongly associated with the ability to accurately perceive activity in the body (interoception: Craig, 2009; see Critchley Wiens, Rotshtein, Öhman, & Dolan, 2004). In other words, the generation and perception of bodily responses may be an important mechanism through which emotion drives rejection on the UG.

However, the direction of the relationship between bodily responses and rejection rates cannot yet be inferred. In particular, it is currently unclear whether these bodily responses are simply downstream consequences of brain-based emotion regulation processes that play no active part in UG rejection, or whether bodily reactions are a central part of the mechanism that shapes decisions to reject or accept. If bodily responses are simply epiphenomena, they cannot genuinely explain individual differences in UG behavior.

These bodily feedback accounts are notoriously difficult to test with causal methodologies, because of the difficulties in fully isolating the brain from the body or in simultaneously manipulating all of the relevant bodily feedback systems (see Dunn et al., 2006). We have previously argued that, in the absence of tractable causal methodologies, some evidence for the direction of the relationship between bodily responses and cognitive–affective processes can be gleaned from moderation approaches. In particular, a prediction arising from Jamesian bodily feedback theories is that activity in the body should more strongly influence cognitive–affective processing in those individuals that can accurately listen in to it (“interoception”: see Dunn, Galton, et al., 2010a; Werner, Jung, Duschek & Schandry 2009). Supporting this prediction, those with superior interoceptive ability showed a greater coupling between HR responses and self-reported arousal (but not valence) in response to emotional stimuli (Dunn et al., 2010a, Study 1). Furthermore, intuitive decision making was more strongly influenced by bodily responses, both for better and for worse, in those with accurate interoception. Where individuals exhibited greater bodily responses to unprofitable relative to profitable options, accurate interoception aided decision making. In contrast, where bodily responses were more marked for profitable than for unprofitable options, accurate interoception impaired decision making (Dunn et al., 2010a, Study 2). In our view, these findings can be straightforwardly accommodated only by a model that gives bodily signals a partly causal, rather than solely epiphenomenal, role. If the body is simply a downstream consequence of the response, then why would the degree to which one can tune in to the body shape how strongly it is coupled to thinking and feeling?

The primary aim of the present study was to test whether interoceptive ability also moderates the relationship observed between bodily responses and social decision making on the UG. In particular, if this moderation account is correct, we would expect that bodily responses would be unrelated to rejection behavior in those with poor interoception, but that a greater bodily response to rejected relative to accepted offers would be associated with greater behavioral rejection rates in those with accurate interoception. If such a moderation relationship is found, this would provide evidence that bodily signals (or “gut feelings”) partly underpin emotionally driven rejection behavior. Moreover, it would suggest the interplay between emotional bodily responses and their perception can account for a share of individual variation on the UG. As far as we are aware, no studies have yet looked at how interoception relates to UG rejection. Although Kirk et al. (2011) raised the theoretical possibility that individual differences in interoceptive ability relate to behavior on the UG, they did not include a behavioral measure of interoception nor any physiological indices of bodily response.

To address this question we administered a version of the UG previously used in psychophysiology studies (van’t Wout et al., 2006), recording EDA and HR responses while participants considered each offer. Previous psychophysiological studies of the UG have differed in whether they primarily focus on bodily responses to fair versus unfair (Moretti et al., 2009; van’t Wout et al., 2006) or accepted versus rejected (Osumi & Ohira, 2009) offers. In the present study, we concentrated on the differential response to offers that were rejected versus accepted, since this mostly closely maps onto our behavioral measure (i.e., proportion of offers rejected). To index interoception accuracy, we utilized the mental tracking task (Schandry, 1981), which asks individuals to count their heartbeats and compares these judgments to the electrocardiogram record.

We also measured heart rate variability (HRV) at rest prior to the UG. Increasing HRV reflects the degree to which cardiac activity can be adjusted by the brain to meet changing environmental demands. Such regulation is believed to be implemented in part via parasympathetic (vagal) efferent control mechanisms and is increasingly viewed as a good measure of trait emotion regulation capability (see Appelhans & Leucken, 2006). For example, reduced vagus influence on the heart may promote mobilization behaviors such as fight or flight, and, conversely, increased vagal influence should result in social engagement behaviors (see Porges, 1995). Similarly, vagal inhibitory mechanisms are seen as a central form of emotional control in the theory of neurovisceral integration (Thayer & Lane, 2000). The brain both controls the heart via projections to the vagus from preganglionic sympathetic and parasympathetic neurons and receives input signals from the heart via the baroreceptor reflex. Consistent with these frameworks, greater HRV has previously been associated with reduced rejection rates on the UG in some individuals (Harlé, Allen, & Sanfey, 2010). This can be understood as increasing vagal control of the heart promoting affiliative behavior (i.e., offer acceptance).

Our first hypothesis was that there would be greater electrodermal response and more marked HR responses to rejected, relative to accepted, offers, in line with previous psychophysiological studies of the UG (Osumi & Ohira, 2009). Our second (and central) hypothesis was that interoception would moderate the degree to which bodily emotional responses correlated with overall rejection rates of unfair offers. For those with accurate interoception, we expected greater bodily responses to rejected relative to accepted offers to predict greater rejection behavior. However, in those with poor interoception, we predicted that bodily responses would be unrelated to rejection behavior. Our third hypothesis was that higher HRV variability (indicating greater ability to centrally regulate the function of the heart via the vagus) would predict fewer rejections of offers. This is based on the polyvagal perspective that greater control of the heart encourages affiliative behavior, as opposed to fight or flight responding (see Porges, 1995). We had no a priori hypothesis that interoception would moderate the relationship between HRV and rejection rates. However, we examined this possibility in exploratory analyses.

## Method

### Participants

Fifty-one healthy participants (36 female; mean age = 37.81 years, *SD* = 17.07, range = 18–65) completed the UG and the interoception task. All of the participants were in the normal IQ range (mean = 118.15, *SD* = 8.81, range 93.40–130.60), which was estimated using the National Adult Reading Test (Nelson, 1982). Age, gender, and IQ were not significantly related to overall, computer, or human rejection rates (greatest *r* = .23; smallest *p* = .10). The study was approved by the Cambridge University psychology research ethics committee, and all participants gave written informed consent prior to taking part. Volunteers were given an honorarium of £6 per hour for their time and were awarded £3 to cover travel expenses.

### Ultimatum game

The UG was as described in (Dunn, Makarova et al., 2010b). Participants played the role of responder in 20 one-shot UGs offering a division of £10. There were 10 fair (£5), two slightly unfair (£3), four moderately unfair (£2), and four very unfair (£1) offers, presented in a random sequence. Participants were told that 50 % of each offer type was proposed by previous participants, and the other half was generated randomly by the computer **(**in reality, the offers were determined by the experimenter and were fixed across all participants). On each trial, a fixation cross was displayed, the proposer or the computer was shown, and the offer was presented (10 s each). Participants then responded to the proposal, before feedback was delivered for 10 s. As a manipulation check, after completing all 20 UG trials, participants retrospectively rated how angry each offer type made them feel and how fair each offer was, on a scale from 0 (*not at all*) to 100 (*extremely*). We chose not to take anger and fairness ratings immediately after each trial, because we did not want these ratings to influence subsequent rejection behavior. Prior to the responder trials, all of the participants also made five proposals for future players on the task and had their photographs taken. This approach was taken to make it believable that the human offers that participants received were made by real people. To keep participants highly motivated, they were instructed that one of their twenty responder decisions would be chosen at random to be paid out on, meaning their decisions were not purely hypothetical.

### Interoception task

Interoceptive accuracy was measured using the Schandry (1981) heartbeat perception task as described in (see Dunn et al., 2010a, 2010b). On each of six computerized trials, participants counted how many heartbeats they felt over a period of time (2 × 35 s, 2 × 25 s, and 2 × 45 s), and this was compared to how many heartbeats occurred on a simultaneous ECG trace. To control for the possibility that participants performed the mental tracking task by counting time and then making an educated guess according to underlying beliefs about HR (Ring & Brener, 1996), we took two steps. In three time-estimation trials (1 × 23 s, 1 × 56 s, 1 × 40 s in length) participants were asked to count how many seconds elapsed between two tones, with estimates being compared to a stopwatch recording. Additionally, resting HR during a 3-min relaxation period prior to the interoception task was measured and then compared with participants’ estimate of their resting HR to index HR belief accuracy (calculated in the same way as heart and time estimation accuracy). If the time approximation strategy is confounding results, any relationship between interoceptive accuracy and UG behavioral and physiological measures should no longer hold when covarying out these variables. Age, gender, body mass index (BMI), resting HR, IQ, and physical activity (assessed using the scale described by Ehlers & Breuer, 1992) have also previously been associated with heartbeat perception accuracy, so these details were additionally measured and entered as covariates.

Interoception accuracy, time accuracy (on a trial-by-trial basis), and HR belief accuracy were expressed as percentage error scores. Following the standard approach in the literature, these were calculated by taking the modulus of the actual value minus the estimated value, dividing this by the actual value, and then multiplying by 100 ([∣actual–estimated∣÷actual ] × 100) (see Ehlers & Breuer, 1992). Modulus scores were used as the primary index of accuracy; the distribution of nonmodulus scores is very difficult to interpret, because very negative and very positive values would both indicate poor accuracy. In practice, nearly all participants underestimated their HRs in the present sample, meaning that the modulus and nonmodulus scores were extremely similar (*r* = .98, *p* < .001).

We selected the mental tracking task (Schandry, 1981) rather than alternative tone detection procedures (e.g., Whitehead, Drescher, Heiman, & Blackwell, 1977) to measure heartbeat perception since, when piloting the latter in our laboratory, we found they were not a particularly sensitive individual differences measure. Specifically, a large proportion of participants performed at chance levels, consistent with the relatively low rates (40–50 % classified as perceivers) of detection typically found in the literature (e.g., Eichler & Katkin, 1994; Knapp-Kline & Kline, 2005), introducing a floor effect into the data and reducing the sensitivity of the task as an individual differences measure. There is no clear superiority for either task in terms of how well they deal with possible interpretation confounds (Knapp-Kline & Kline, 2005) or how widely they are used in the literature (see Dunn et al., 2010a, 2010b supplementary materials). Given that the primary focus of the present study was on individual differences, we therefore selected the Schandry task.

### Psychophysiology recording

EDA (in microsiemens; μS) and HR (in beats per minute; BPM) responses when receiving each offer were recorded using a BIOPAC MP100 system (BIOPAC, 1997) acquiring data at 1,000 samples per second. We conceptualized these parameters as measures of core affect rather than discrete emotions (cf. Barrett, 2006; Barrett et al., 2004; Dunn et al., 2010a, 2010b, Study 2), with EDA particularly relating to sympathetic nervous system modulation of arousal/activation and HR as particularly relating to joint sympathetic and parasympathetic modulation of valence (e.g., Bradley, Codispoti, Cuthbert, & Lang, 2001). EDA was recorded using two grounded Ag–AgCl electrodes (BIOPAC TSD203 transducer) that were secured ventrally on the distal index and middle finger of the nondominant hand, with BIOPAC EDA paste (with a NaCl concentration of 0.05 M) as the electrolyte. Two disposable Ag–AgCl ECG electrodes were placed on the dorsal forearms with clip-on shielded leads attached for HR recording.

Data were averaged into half-second chunks prior to analysis. HR responses were quantified as mean HR change during the offer period, relative to a pre-offer 1-s baseline. (see Dunn et al., 2010a). EDA responses were quantified as the maximum positive change observed during the 6-s offer period, excluding trials where no positive change occurred (i.e., amplitude; cf. Pollatos, Schubö, Herbert, Matthias, & Schandry, 2008).^2^

We computed the median value of EDA and HR response to each offer type and additionally natural log transformed the EDA variables, prior to analysis to minimize outlier effects. Since the EDA analysis did not use a baseline subtraction to control for variation in background activity, we covaried mean EDA across the entire UG in all individual differences analyses.

We also recorded HR variability during a 5-min baseline recording taken prior to the UG as an additional measure of trait emotion regulation. A tachogram of the R–R intervals for each participant was visually inspected for marked outliers. Where outliers were identified, the raw ECG data was reexamined and adjusted if necessary. HRV was then indexed using CMetX software (Allen, Chambers, & Towers, 2007). We analyzed log HRV as a measure of overall HRV, the Cardic Vagal Index (CVI) as a measure of vagal (parasympathetic) contribution to HRV, and the Cardiac Sympathetic Index (CSI) as a measure of sympathetic contribution to HRV.

## Results

### Behavioral data

**Fig. 1:**
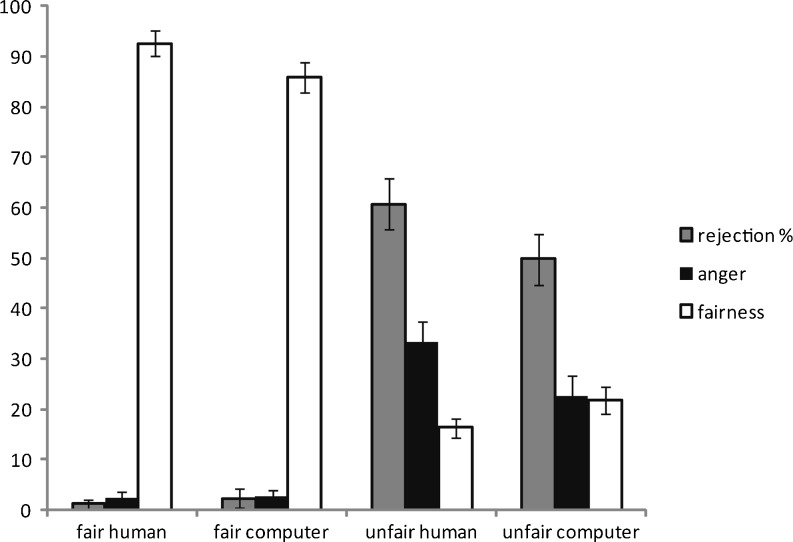
Percentage of offers rejected, anger ratings and fairness ratings for each offer type. Anger and fairness ratings on scales from 0 (*not at all*) to 100 (*extremely*). Data are mean (standard error of the mean) values

Figure 1 plots rejection percentage, fairness ratings, and anger ratings for each offer type. Showing that the task was working as intended, participants rejected more unfair than fair offers, *F*(1, 50) = 139.05, *p* < .001, and tended to reject more human than computer offers, *F*(1, 50) = 3.21, *p* = .08. There was an interaction between fairness and proposer type, *F*(1, 50) = 5.23, *p* = .03, with participants rejecting more unfair offers from human proposers than computer proposers, *t*(50) = 2.13, *p* = .04, but not differing in their rejections of fair human or computer offers, *t* < 1. The use of ANOVA approaches with proportion data has been criticized, and alternative multilevel logistic regression techniques have been recommended (Jaeger, 2008). Multilevel analyses consider effects at the individual trial level, taking into account that each trial is nested within a particular participant. We repeated the key rejection rate analysis using multilevel logistical regression (applying the xtmelogit command in Stata 11.0; StataCorp, 2009). Rejection rates were greater for unfair than for fair, B = 5.08, *SE* = 0.61, *Z* = 8.32, *p* < .001, and for human than for computer, B = .65, *SE* = .22, *Z* = 2.97, *p* < .01, offers. However, in contrast with the ANOVA analyses, there was no significant interaction between fairness and proposer, B = 1.28, *SE* = .84, *Z* = 1.52, *p* = 0.13.

More anger was experienced to unfair than to fair offers, *F*(1, 50) = 62.86, *p* < .001, and to human than to computer offers, *F*(1, 50) = 10.04, *p* < .04, and again there was an interaction between fairness and proposer, *F*(1, 50) = 10.91, *p* < .01. Participants felt more angry to unfair human offers than to unfair computer offers, *t*(50) = 3.24, *p* < .01, but did not differ for human and computer fair offers, *t* < 1. Participants rated unfair offers as less fair than fair offers, *F*(1, 50) = 424.51, *p* < .001. Although there was no overall difference between fairness ratings of human and computer proposals, *F* < 1, there was an interaction between fairness and proposer, *F*(1, 50) = 10.99, p < .01. Participants reported that human unfair offers were less fair than computer unfair offers, *t*(50) = 2.65, *p* = .01, but that human fair offers were more fair than computer fair offers, *t*(50) = 3.09, *p* < .01. Since anger and fairness ratings were taken retrospectively rather than after each offer, it was not possible to conduct multilevel analyses. We subsequently focus on rejection rates, but for further analysis of the anger and fairness responses see the Supplementary Material.

### Psychophysiology responses to offers

**Fig. 2:**
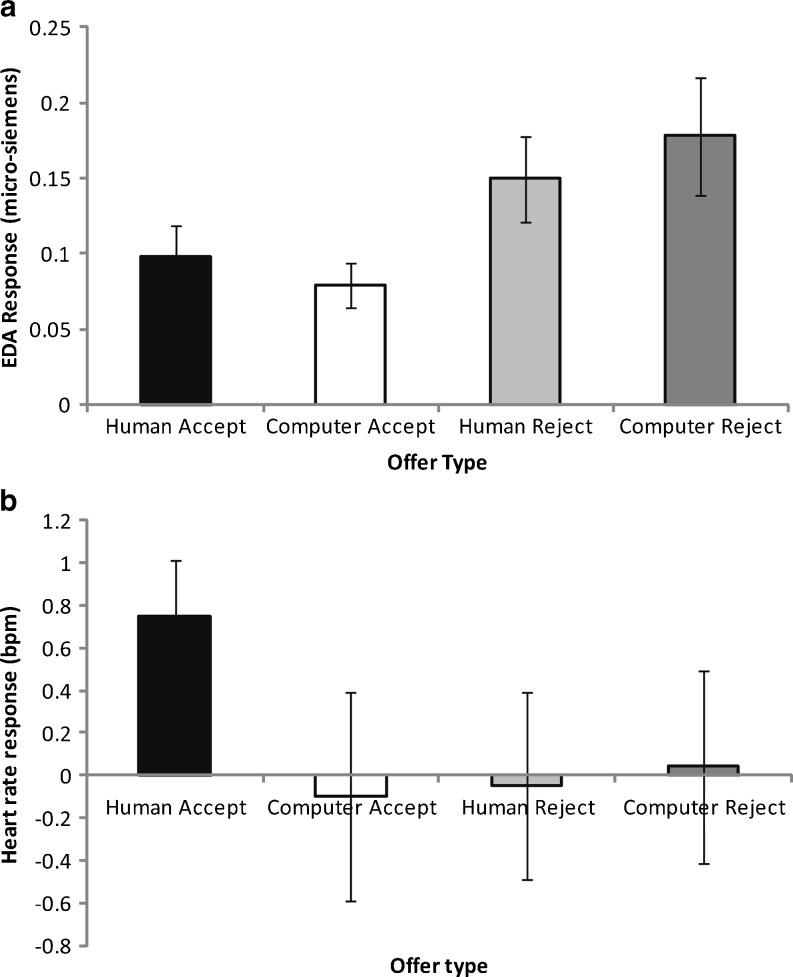
EDA (**a**) and HR (**b**) responses to different offer types on the UG. Data are mean (standard error of the mean) values

Thirty-two participants both rejected and accepted human and computer offers. There was a greater EDA response to rejected than to accepted offers, *F*(1, 31) = 14.19, *p* = .001, but there was no main or interaction effect of proposer, *F*s < 1 (see Fig. 2a). Comparable analyses were conducted on the HR data, excluding two outliers with significant movement artefact in their recording trace. There were no main effects of proposer or decision, *F*s < 1, and the interaction was nonsignificant, *F*(1,31) = 1.75, *p* = .20 (see Fig. 2b). These results support Hypothesis 1 for the EDA but not HR data.^3^

Because there were no significant proposer effects and we had no a priori hypotheses regarding differential bodily responses as a function of proposer, for subsequent analyses, we collapsed across human and computer offers to increase the sample size available to analysis. Forty-five participants both rejected and accepted offers. Offers that were rejected continued to produce a greater response than those that were accepted in terms of EDA, *t*(44) = 2.95, *p* < .01, but not HR, *t*(44) = 1.63, *p* = .11.

Next, we examined whether individual differences in bodily responses predicted UG rejection behavior. We subtracted EDA/HR responses to accepted from rejected offers, with a larger value on this differentiation index indicating a greater response to rejected, relative to accepted, offers. There was no significant relationship between overall rejection rates and EDA differentiation (covarying for mean EDA level), *r*_*p*_ = .01, *p* = .72, or HR differentiation, *r* = –.22, *p* = .15.

### Moderating role of interoception

Of the 45 individuals with EDA responses to both accepted and rejected offers, one had incomplete interoception data, and three had incomplete rest data because of experimenter error, leaving a sample of 41 available for analysis. There was a good spread of interoceptive ability across the sample (mean error score = 26.51 %, *SD* = 13.61, range = 1.56–57.65). In zero-order correlations, interoception error was not significantly related to overall rejection rates, EDA differentiation, or HR differentiation, *p*s > .17. However, interoception error was trend related to reduced anger, *r* = –.25, *p* = .09, and was significantly related to less unfair fairness, *r* = .34, *p* = .02, ratings of unfair (relative to fair) offers. In other words, as individuals’ interoceptive abilities improved they showed a greater anger response to unfair offers and experienced inequitable offers as more unfair.

To examine whether interoception moderated any relationships between UG behavior and bodily responses (Hypothesis 2), we conducted a series of multiple regression analyses. The proportion of rejections was the dependent variable. At step one of the regression, we entered interoception error, EDA differentiation, and mean EDA during the UG (all *z*-scored). At the second step, we entered the product term of the interoception error and EDA differentiation variables (Aiken & West, 1991; Baron & Kenny, 1986). Significant moderation is indicated by the fit of the model improving from step one to step two. Comparable analyses were conducted for HR differentiation, except that mean HR during the UG was not included as an additional covariate.

We examined each of these analyses for the presence of multivariate outliers using Mahalanobis distance. Following Tabachnik and Fidell (2001), we identified the χ^2^ value that would be significant at the *p* < .001 level for analyses with degrees of freedom equal to the number of independent variables (four for EDA; three for HR). All data points greater than each of these values were excluded. This process was repeated iteratively until no outliers remained. Three outliers were excluded on this basis, leaving a final sample size of 38.

**Fig. 3:**
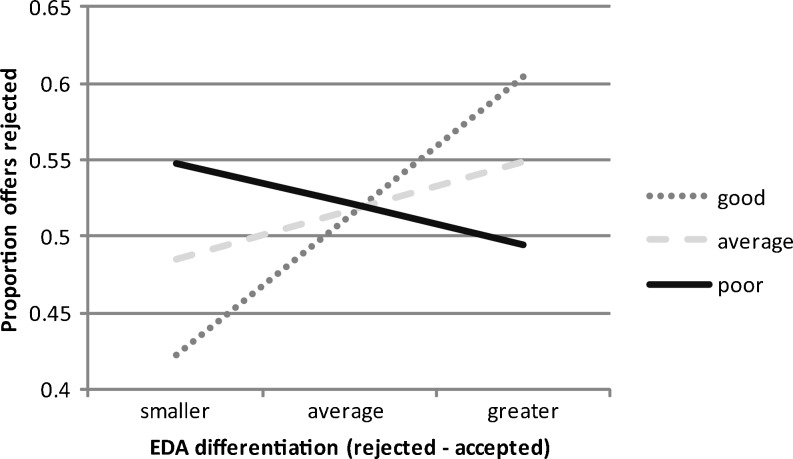
The relationship between EDA differentiation and the proportion of human offers rejected as a function of good, average, and poor interoceptive accuracy. EDA differentiation = response to rejected minus accepted offers. More negative EDA differentiation = –1 *SD*; average EDA differentiation = 0 *SD*; more positive EDA differentiation = +1 *SD*. Good interoception = –1 *SD* error score; average interoception = 0 *SD* error score; poor interoception = +1 *SD* error score

In the EDA analyses, a significant moderating role of interoception was observed, Δ*F*(1, 33) = 7.05, *p* = .01, Δr^2^ = .15, *r* ^2^ total = .28. Figure 3 plots this interaction using the method of simple slopes (Aiken & West, 1991; see Dunn et al., 2010a, for a full description of this approach). Consistent with Hypothesis 2, greater EDA differentiation predicted higher rejection rates in those with better interoception, whereas there was no clear relationship between rejection levels and EDA response in those with poorer interoception. The interaction remained significant if ranking data prior to the analysis (Conover & Iman, 1981) to further minimize outlier effects, Δ*F*(1, 35) = 8.38, *p* < .01, Δ*r* ^2^ = .18, *r* ^2^ total = .29. The interaction also held if additionally controlling for all of the possible nuisance variables that have been linked to interoceptive awareness in previous research (entering age, gender, estimated IQ, physical activity, time estimation error, HR belief accuracy, and BMI at step one of the regression), Δ*F*(1, 23) = 14.38, *p* < .001, Δ*r* ^2^ = .21, *r* ^2^ total = .66. This suggests that performance on the cardiac perception task is genuinely measuring interoceptive awareness and is not acting simply as a proxy for other variables such as age, IQ, and so on. In HR analyses (excluding two multivariate outliers), there was no significant moderating role of interoception, Δ*F* < 1. The reported effects were unchanged when the rejection proportion variables were transformed using either arcsine or logit transformations following Jaeger (2008).

### Heart rate variability and UG rejection

Five participants HRV data could not be corrected for movement artefact, leavening a sample of 46 participants for HRV analysis. Consistent with Hypothesis 3, increasing HRV was related to total offers rejected for both log HRV, *r* = –.35, *p* = .02, and the cardiac vagal index (CVI), *r* = –37, *p* = .01, a measure of sympathetic contribution to HR regulation. There was no significant relationship with the CSI, *r* = .13, *p* = .38. This suggests it is primarily parasympathetic mechanisms that are related to UG rejection rates.

**Fig. 4:**
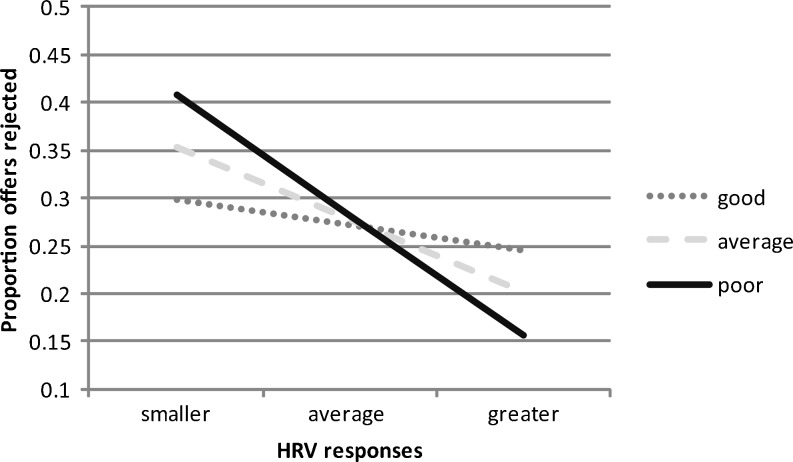
The relationship between HRV and the proportion of human offers rejected as a function of good, average, and poor interoceptive accuracy. Good interoception = –1 *SD* error score; average interoception = 0 *SD* error score; poor interoception = +1 *SD* error score

We also conducted exploratory analyses to establish whether interoceptive awareness moderated the link between HRV and UG responses. A trend significant moderating role of interoception was found on the relationship between log HRV and rejection rates, Δ*F*(1, 41) = 3.45, *p* = .07, Δ*r* ^2^ = .07, *r* ^2^ total = .20 (see Fig. 4). Greater HRV predicted lower rejection rates in those with worse interoception, but was unrelated to rejection rates in individuals with better interoception. This finding again held if additionally controlling for the nuisance variables previously related to cardiac perception performance (entering age, gender, estimated IQ, physical activity, time estimation error, HR belief accuracy, and BMI at step one of the regression). An identical pattern of findings emerged if using the CVI index of HRV, but no findings were significant if using the CSI measure. This further indicates it is the vagal (parasympathetic) component of HRV that is largely accounting for these results.

## Discussion

Rejection behavior on the UG has been understood as a failure of emotion regulation. Here, we attempted to extend this account by examining whether the bodily component of emotional responses can account for individual variability in rejection rates (see bodily feedback theories; Craig, 2009; Damasio, 1994; James 1884). Showing that the UG functioned as expected in the present study, participants rejected more unfair offers, experienced greater anger to unfair offers, and rated them as less fair. This further indicates that rejection of unfair offers and the subjective responses to unfairness on the UG are robust phenomena. Individuals exhibited a greater EDA response to rejected, relative to accepted, offers, as predicted in Hypothesis 1. However, there was no difference in HR responses to accepted versus rejected offers. Although rejection rates, anger ratings, and unfairness ratings were greater for human relative to computer offers, there was no significant difference in bodily responses as a function of proposer. This suggests that enhanced behavioral reaction to unfair human (relative to computer) proposals is not driven solely by bodily feedback mechanisms, and may instead reflect some other, as yet unspecified, mechanisms.

That we observed some effects for EDA but not HR parallels mixed results in the existing literature. For example, although van’t Wout et al. (2006) and Moretti et al. (2009) found that EDA differentiated between fair and unfair offers in healthy participants, Osumi and Ohira (2009) reported that HR but not EDA related to offers that were rejected versus accepted. It is noteworthy that the present study has a larger sample than these earlier studies (*N* = 51 as compared with *N* = 20 in Osumi and Ohira, 2009; *N* = 30 in van’t Wout et al., 2006; and *N* = 14 in Moretti et al., 2009), so our null HR results are unlikely to reflect lack of power. Moroever, similar negative findings emerged when looking at initial HR deceleration, subsequent HR acceleration, and variability in HR response during the offer period, meaning this pattern of findings is not an artefact of the particular analysis strategy we adopted. Changes in mean HR activity are difficult to unambiguously interpret in any case, because of the difficulty in disentangling sympathethic and parasympathetic contributions to HR control. In contrast, EDA provides a relatively pure measure of sympathetic nervous system function.

Our second (and central) hypothesis that interoceptive accuracy would moderate the relationship between bodily responses and rejection rates, was supported for EDA data only. We found a significant moderating role of interoception on the relationship between EDA responses and rejection rates of offers. Those with accurate interoception showed a positive coupling between increased unfair rejection rates and greater EDA responses to rejected, relative to accepted, offers. This association between bodily responses and rejection rates largely disappeared in those with inaccurate interoception. This is consistent with emotion regulation accounts of UG rejection behavior (e.g., Sanfey et al., 2003), extending these by showing that embodied components of emotional reactions also drive responses to financial inequity.

These results conceptually replicate our previous findings that interoception moderates the relationship between bodily responses and cognitive–affective processes (Dunn et al., 2010a), this time in the social decision-making domain. This extends previous psychophysiological findings on the UG (e.g., van’t Wout et al., 2006) by showing that it is the *interplay* between bodily responses *and* their perception that can best account for individual differences in rejection rates. It is unsurprising that no moderation effect emerged for the HR data, given that HR response did not differentiate between rejected versus accepted offers in the first place.

The present data speak to the direction of the relationship between bodily changes and cognitive–affective processes observed in earlier studies. Jamesian theories would directly predict that interoceptive accuracy will moderate the extent to which bodily responses shape how we think and feel (e.g., Damasio, 1994; James, 1884; see Dunn et al., 2010a). However, accounts that view bodily responses solely as an epiphenomenom would not predict a priori a moderating role of interoception and struggle to account for this finding parsimoniously. In particular, if the body is nothing but a downstream consequence of a central brain-based response, then why would the accuracy with which an individual can monitor the body determine the degree of coupling between bodily responses and cognitive–affective processes? For this reason, we feel that the present results are more easily explained by accounts suggesting bodily responses are at least partly causal rather than solely epiphenomenal.

It was further observed that greater HRV (particularly parasympathetic components) was associated lower rejection rates across the sample, as predicted in Hypothesis 3. Given that HRV is viewed as measure of dispositional emotion regulation capacity (Appelhans & Leucken, 2006), this further supports the hypothesis that rejection behavior on the UG relates to a failure in emotion regulation processes, as evidenced for example by increased rejection in patients with ventromedial prefrontal cortex damage (Koenigs & Tranel, 2007). In particular, vagal inhibitory mechanisms are seen as central in allowing individuals to flexibly adjust metabolic expenditure in rapidly changing social environments (Porges, 1995). As the vagus exerts greater control on the heart, this reduces flight/fight behavior and instead promotes affiliative responses. Acceptance of UG offers can be viewed as an example of such affiliative tendencies at the individual levels (although see accounts arguing that rejection is a prosocial behavior at the societal level, since it punishes behavior that violates group norms; Fehr & Fischbacher, 2003).

In exploratory analyses, we found that interoceptive awareness mediated the link between HRV and UG responses. Contrary to the state EDA findings, there was a nonsignificant trend for greater HRV to become increasingly associated with lower rejection rates as interoceptive accuracy *worsened*. One way to interpret this pattern of findings is to propose that trait HRV and state EDA have independent effects on the UG. In poor interoceptors, state EDA changes have little impact because the individual cannot accurately detect them, so UG decision making is more strongly influenced instead by trait HRV. In contrast, in accurate interoceptors, state EDA has a large impact as the individual is sensitively attuned to them, so UG decision making is less clearly affected by trait HRV. Again, this moderation relationship is difficult to explain with frameworks that argue that bodily responses are not causally involved in the decision-making process. As examining the interaction between HRV and interoception was not an a priori aim of this study and the moderation effect was only trend significant, these results require replication and should be interpreted cautiously at the present time. That EDA primarily reflects sympathetic nervous system function, whereas the HRV effects are primarily parasympathetic in nature, is consistent with the notion that they will have independent effects on UG decision making.

Overall, these findings also suggest a possible reinterpretation of what insular activation found in previous fMRI studies during the UG actually means. Given that the anterior insular has been robustly implicated in interoception (Craig 2009; Critchley et al., 2004), it is plausible that previous findings of insular activation on the UG might reflect the general representation of bodily responses (see also Kirk, Downar and Montague 2011), as opposed to activation of distinct negative affective states such as disgust or anger (Sanfey et al., 2003). Indeed, many functional imaging studies using appetitive tasks also detect anterior insula responses, particularly for monetary wins that are also physiologically arousing (Clark, Crooks, Clarke, Aitken, & Dunn, 2012; Clark, Lawrence, Astley-Jones, & Gray, 2009; Elliot, Friston, & Dolan, 2000), indicating that the insular is not specific to negative emotions. In our view, the existing evidence is most consistent with the view that bodily signals provide a crude sense of emotional arousal (i.e., core affect; Barrett, 2006), which needs to be appraised centrally to lead to clearly valenced emotions or discrete emotional states (Barrett et al., 2004; Dunn et al., 2010a, Study 1; Schachter & Singer, 1962). This reinterpretation of insular function on the UG of course relies on reverse inference, and it would now be useful for a combined fMRI and psychophysiology study to be run to test this account.

It is important to be clear that we are not claiming that central brain-based mechanisms are *not* involved in social decision making. In particular, bodily responses have to be generated in the first place, presumably by some kind of activity in the brain. Nor do our findings indicate that bodily responses are a necessary component of the mechanism underpinning UG rejection. In particular, those with poor interoception nevertheless rejected the same proportion of offers as those with accurate interoception. This further indicates there are individual differences in the degree to which embodiment mechanisms are implicated in cognitive–affective processing (Dunn et al., 2010a).

An important question to consider is whether the present findings have any implications for everyday decision making. As previously discussed, rejection behavior on the UG is “irrational” in the sense that it leads to personal financial loss (cf. emotional dysregulation accounts; Sanfey et al., 2003), but is “rational” in light of the potential benefits for the social groups in enforcing social norms (cf. Fehr & Fishbacher, 2003). State increases in electrodermal reactivity to financial offers, and enhanced perception of these changes via interoceptive mechanisms, appear to lead to societal gain but personal cost (i.e. greater rejection of unfair offers). Trait regulation of the body via increased HRV arguably leads to personal gain but brings costs to the group (i.e. reduced rejection of unfair offers).

It is possible that a range of training techniques could develop this flexibility and therefore promote adaptive social decision making. For example, helping individuals to regulate HRV could allow them to decide if they wish to reduce “rejection” behavior by increasing vagal control or increase “rejection behavior” by relinquishing vagal control. Moreover, training the capacity to modify interoception, perhaps by use of neurofeedback of right anterior insular activity (e.g., Caria et al., 2007), could enable individuals to tune in or out of bodily signals as required. Finally, regular bodily focused meditation practices (Sze, Gyurak, Yuan, & Levenson, 2010) could change the coupling seen between bodily responses and subsequent rejection behavior. In particular, the observing, nonjudgmental relationship to the body that mindfulness cultivates may facilitate more reflective and less impulsive reactions to “gut feelings.” Consistent with this possibility, expert meditators have been found to activate the posterior rather than anterior insular when performing the UG (Kirk et al., 2011), and it has been argued that this shift accounts for their decreased rejection rates of unfair offers. Of course, these intervention ideas are speculative at the present time and require empirical validation.

There are a number of limitations of the present study that need to be held in mind. First, we focused only on cardiac interoception. We chose the Schandry task because it is viewed as a general index of interoceptive awareness and has been most closely linked to cognitive–affective processes (see Dunn et al., 2010a). Nevertheless, it is possible that noise has been introduced into the present data set by measuring interoception in the cardiac domain and relating this to bodily responses to the UG in the electrodermal domain. By incorporating multiple measures of bodily response and perception this potential source of error can be minimized in future work. Second, we indexed only EDA and HR. Although this is appropriate given the present focus on arousal, it is not sufficient to make strong claims about whether or not bodily responses lead to distinct emotions such as anger and disgust (cf. Larsen et al. 2007). Third, although a robust measure of social decision making, the UG is nevertheless of questionable ecological validity. It would now be interesting to see whether comparable results emerge in more real-world contexts. Fourth, although the interplay between bodily responses, their perception, and their regulation did account for significant individual variation in rejection rates, a large amount of the variance in the model nevertheless remained unexplained. This suggests other additional factors need to be examined (e.g., individual differences in social altruism or trait affect; see Dunn et al., 2010b; Fehr & Fishbacher, 2003). Fifth, the moderation approach adopted here, although suggestive of the direction of the relationship between body and mind, does not provide conclusive causal support for bodily feedback theories. In particular, an alternative explanation of the interaction effects is that bodily responses moderate the response between rejection rates and interoception. To definitively establish the causal role of bodily feedback mechanisms, it is necessary to manipulate interoception and/or bodily response in future work. Sixth, we utilized retrospective anger and fairness ratings of the offers, because we did not want these judgments to bias, reject, or accept decisions. However, this is likely to have reduced their sensitivity.

In conclusion, the present data further support the notion that emotion regulation mechanisms relate to rejection behavior on the UG. Our results suggest that “gut feelings” arising from the body, interacting with the ability to accurately perceive bodily feedback, partly shape social decision making. Furthermore, superior capacity to regulate bodily responses via vagal inhibition (indexed in terms of HRV) also influences the degree to which individuals respond emotionally on the UG. This interplay between bodily response, regulation and perception can therefore account for individual differences in how we react to perceived unfairness.

## Footnotes

1 It is debatable whether emotionally driven rejection behavior on the UG is necessarily “illogical” in nature. From a purely selfish economic perspective, rejection is irrational since it leads to financial loss. However, from a social perspective, rejection can be seen as an altruistic action to preserve social norms. Rather than maximizing self-interest, the responder is punishing socially inappropriate action from the proposer for the good of the general population (Fehr & Fischbacher, 2003; Fehr & Gaecter, 2002). In this sense, the strong emotional reactions to unfairness could adaptively function to trigger punishment of the proposer (Harmon-Jones & Siegelman, 2001). Similarly, rejection of unfair offers could adaptively maintain positive self-esteem (Dunn et al., 2010a, 2010b).

2 Participants displayed EDA responses to 15 out of 20 trials on average (mean nonresponses = 4.25, *SD* = 3.48). To examine whether the proportion of nonresponses varied as a function of offer type, a repeated measures ANOVA was conducted with fairness (fair, unfair) and proposer (human, computer) as within-subjects factors. There were no significant main or interaction effects, *p*s > .09, indicating response rates are comparable across offer types.

3 There were also no significant HR effects if looking at maximum deceleration in the first 3 s, maximum acceleration in the second 3 s (cf. Dunn et al., 2010a, 2010b, Study 2), or HR variability during the 6-s offer period (the standard deviation of R–R intervals), *p*s > .05. For the sake of completeness, we also analysed psychophysiology responses as a function of whether the offer was fair or unfair (cf. van’t Wout et al., 2006). Greater EDA responses were exhibited to unfair than to fair offers, *F*(1, 49) = 9.29, *p* < .01, but there was no main or interaction effect of proposer, *F*s < 1. Identical analyses were conducted on the HR data, excluding three outliers. There was no main effect of fairness, *F*(1, 48) = 1.14, *p* = .29, or proposer, *F*(1, 48) = 1.05, *p* = .31, and no interaction between fairness and proposer, *F* < 1.

## References

[CR1] Aiken LS, West SG (1991). Multiple regression: Testing and interpreting interactions.

[CR2] Allen JJB, Chambers AS, Towers DN (2007). The many metrics of cardiac chronotropy: a pragmatic primer and a brief comparison of metrics. Biological Psychology.

[CR3] Appelhans BM, Luecken LJ (2006). Heart rate variability as an index of regulated emotional responding. Review of General Psychology.

[CR4] Baron RM, Kenny DA (1986). The moderator–mediator variable distinction in psychological research: conceptual, strategic, and statistical considerations. Journal of Personality and Social Psychology.

[CR5] Barrett LF (2006). Are emotions natural kinds?. Perspectives on Psychological Science.

[CR6] Barrett LF, Quigley KS, Bliss-Moreau E, Aronson KR (2004). Interoceptive sensitivity and self-reports of emotional experience. Journal of Personality and Social Psychology.

[CR7] Bradley MM, Codispoti M, Cuthbert BN, Lang PJ (2001). Emotion and motivation I: defensive and appetitive reactions in picture processing. Emotion.

[CR8] BIOPAC (1997). BIOPAC MP100 Psychophysiological Data Acquisition System: BIOPAC Systems, Inc.

[CR9] Caria A, Veit R, Sitaram R, Lotze M, Weiskopf N, Grodd W (2007). Regulation of anterior insular cortex activity using real-time fMRI. Neuroimage.

[CR10] Clark L., Crooks B., Clarke R., Aitken M. R., Dunn B. D. (2012). Physiological responses to near-miss outcomes and personal control during simulated gambling. Journal of Gambling Studies.

[CR11] Clark L, Lawrence AJ, Astley-Jones F, Gray N (2009). Gambling near-misses enhance motivation to gamble and recruit win-related brain circuitry. Neuron.

[CR12] Conover WJ, Iman RL (1981). Rank transformations as a bridge between parametric and nonparametric statistics. American Statistician.

[CR13] Craig AD (2009). How do you feel—now? The anterior insula and human awareness. Nature Reviews Neuroscience.

[CR14] Critchley HD, Wiens S, Rotshtein P, Öhman A, Dolan RJ (2004). Neural systems supporting interoceptive awareness. Nature Neuroscience.

[CR15] Crockett MJ, Clark L, Tabibnia G, Lieberman MD, Robbins TW (2008). Serotonin modulates behavioral reactions to unfairness. Science.

[CR16] Dalgleish T, Dunn BD, Mobbs D (2009). Affective neuroscience: past, present, and future. Emotion Review.

[CR17] Damasio AR (1994). Descartes' error: Emotion, reason, and the human brain.

[CR18] Dunn BD, Dalgleish T, Lawrence AD (2006). The somatic marker hypothesis: a critical evaluation. Neuroscience and Biobehavioral Reviews.

[CR19] Dunn BD, Galton HC, Morgan R, Evans D, Oliver C, Meyer M (2010). Listening to your heart: how interoception shapes emotion experience and intuitive decision-making. Psychological Science.

[CR20] Dunn BD, Makarova D, Evans D, Clark L (2010). “I'm worth more than that”: Trait positivity predicts increased rejection of unfair financial offers. PloS One.

[CR21] Ehlers A, Breuer P (1992). Increased cardiac awareness in panic disorder. Journal of Abnormal Psychology.

[CR22] Eichler S, Katkin ES (1994). The relationship between cardiovascular reactivity and heartbeat detection. Psychophysiology.

[CR23] Elliott R, Friston KJ, Dolan RJ (2000). Dissociable neural responses in human reward systems. Journal of Neuroscience.

[CR24] Fehr E, Fischbacher U (2003). The nature of human altruism. Nature.

[CR25] Fehr E, Gachter S (2002). Altruistic punishment in humans. Nature.

[CR26] Gross J (1998). The emerging field of emotion regulation: an integrative review. Review of General Psychology.

[CR27] Gross, J. J. (Ed.). (2006). *The handbook of emotion regulation*: Guildford Press.

[CR28] Güth W, Schmittberger R, Schwarze B (1982). An experimental analysis of ultimatum bargaining. Journal of Economic Behavior & Organization.

[CR29] Harle K., Sanfey A. G. (2007). Incidental sadness biases social economic decisions in the Ultimatum Game. Emotion.

[CR30] Harlé KM, Allen JJB, Sanfey AG (2010). The impact of depression on social economic decision making. Journal of Abnormal Psychology.

[CR31] Harmon-Jones E, Sigelman J (2001). State anger and prefrontal brain activity: evidence that insult-related relative left-prefrontal activation is associated with experienced anger and aggression. Journality of Personality and Social Psychology.

[CR32] Jaeger TF (2008). Categorical data analysis: away from ANOVAs (transformation or not) and towards mixed logit mixed models. Journal of Memory and Language.

[CR33] James W (1884). What is emotion?. Mind.

[CR34] Kirk U, Downar J, Montague RP (2011). Interoception drives increased rational decision-making in meditators playing the ultimatum game. Frontiers in Neuroscience.

[CR35] Knapp-Kline K, Kline JP (2005). Heart rate, heart rate variability, and heartbeat detection with the method of constant stimuli: slow and steady wins the race. Biological Psychology.

[CR36] Koenigs M, Tranel D (2007). Irrational economic decision-making after ventromedial prefrontal damage: evidence from the ultimatum game. Journal of Neuroscience.

[CR37] Larsen, T. F., Bernston, C. G., Poehlmann, K. M., Ito, T. A., & Cacioppo, J. T. (2007). The psychophysiology of emotion. In J. T. Cacioppo, L. G. Tassinary, & C. G. Bernston (Eds.), *Handbook of psychophysiology*: Cambridge University Press.

[CR38] Moretti L, Dragone D, Di Pellegrino G (2009). Reward and social valuation deficits following ventromedial prefrontal damage. Journal of Cognitive Neuroscience.

[CR39] Nelson HE (1982). National Adult Reading Test (NART) test manual.

[CR40] Osumi T, Ohira H (2009). Cardiac responses predict decisions: an investigation of the relation between orienting response and decisions in the ultimatum game. International Journal of Psychophysiology.

[CR41] Pillutla MM, Murnighan JK (1996). Unfairness, anger, and spite: emotional rejections of ultimatum offers. Organizational Behavior and Human Decision Processes.

[CR42] Pollatos O, Schubö A, Herbert BM, Matthias E, Schandry R (2008). Deficits in early emotion reactivity in alexithymia. Psychophysiology.

[CR43] Porges SW (1995). Orienting in a defensive world: mammalian modifications of our evolutionary heritage. A polyvagal theory. Psychophysiology.

[CR44] Ring C, Brener J (1996). Influence of beliefs about heart rate and actual heart rate on heartbeat counting. Psychophysiology.

[CR45] Russell JA, Barrett LF (1999). Core affect, prototypical emotional episodes, and other things called emotion: dissecting the elephant. Journal of Personality and Social Psychology.

[CR46] Sanfey AG, Rilling JK, Aronson JA, Nystrom LE, Cohen JD (2003). The neural basis of economic decision-making in the ultimatum game. Science.

[CR47] Schachter S, Singer J (1962). Cognitive, social, and physiological determinants of emotional state. Psychological Review.

[CR48] Schandry R (1981). Heart beat perception and emotional experience. Psychophysiology.

[CR49] StataCorp. (2009). Stata Statistical Software: Release 11.

[CR50] Sze JA, Gyurak A, Yuan JW, Levenson RW (2010). Coherence between emotional experience and physiology: does body awareness training have an impact?. Emotion.

[CR51] Tabachnick BG, Fidell LS (2001). Using multivariate statistics.

[CR52] Tabibnia G, Satpute AB, Lieberman MD (2008). The sunny side of fairness: preference for fairness activates reward circuitry (and disregarding unfairness activates self-control circuitry). Psychological Science.

[CR53] Thayer JF, Lane RD (2000). A model of neurovisceral integration in emotion regulation and dysregulation. Journal of Affective Disorders.

[CR54] van't Wout M, Chang LJ, Sanfey AG (2010). The influence of emotion regulation on social interactive decision-making. Emotion.

[CR55] van't Wout M, Kahn RS, Sanfey AG, Aleman A (2006). Affective state and decision-making in the Ultimatum Game. Experimental Brain Research.

[CR56] Werner NS, Jung K, Duschek S, Schandry R (2009). Enhanced cardiac perception is associated with benefits in decision making. Psychophysiology.

[CR57] Whitehead, W. E., Drescher, V. M., Heiman, P., & Blackwell, B. (1977). Relation of heart rate control to heartbeat perception. *Applied Psychophysiology and Biofeedback, 2,* 371–392. 612350

